# A novel broad specificity fucosidase capable of core α1-6 fucose release from *N-*glycans labeled with urea-linked fluorescent dyes

**DOI:** 10.1038/s41598-018-27797-0

**Published:** 2018-06-22

**Authors:** Saulius Vainauskas, Charlotte H. Kirk, Laudine Petralia, Ellen P. Guthrie, Elizabeth McLeod, Alicia Bielik, Alex Luebbers, Jeremy M. Foster, Cornelis H. Hokke, Pauline M. Rudd, Xiaofeng Shi, Christopher H. Taron

**Affiliations:** 1 New England Biolabs, 240 County Road, Ipswich, MA 01938 USA; 2 Department of Parasitology, Leiden University Medical Center, Albinusdreef 2, 2333 ZA Leiden, The Netherlands; 3 NIBRT GlycoScience Group, National Institute for Bioprocessing, Research and Training, Foster’s Avenue, Mount Merrion, Blackrock, Co, Dublin, Ireland

## Abstract

Exoglycosidases are often used for detailed characterization of glycan structures. Bovine kidney α-fucosidase is commonly used to determine the presence of core α1-6 fucose on *N-*glycans, an important modification of glycoproteins. Recently, several studies have reported that removal of core α1-6-linked fucose from *N-*glycans labeled with the reactive *N-*hydroxysuccinimide carbamate fluorescent labels 6-aminoquinolyl-*N-*hydroxysuccinimidylcarbamate (AQC) and RapiFluor-MS is severely impeded. We report here the cloning, expression and biochemical characterization of an α-fucosidase from *Omnitrophica* bacterium (termed fucosidase O). We show that fucosidase O can efficiently remove α1-6- and α1-3-linked core fucose from *N*-glycans. Additionally, we demonstrate that fucosidase O is able to efficiently hydrolyze core α1-6-linked fucose from *N-*glycans labeled with any of the existing NHS-carbamate activated fluorescent dyes.

## Introduction

Asparagine-linked glycosylation (*N-*glycosylation) is an abundant and complex form of post-translational modification of eukaryotic secretory proteins. Common techniques like liquid chromatography (LC) coupled to fluorescent detection (FLR) and/or mass spectrometry (MS), or capillary electrophoresis (CE) with laser-induced fluorescence (LIF) have been used to structurally characterize *N-*glycans. Data from these analyses can be compared to validated mass or mobility data in reference databases to permit assignments of *N-*glycan structures^1,2^. An added measure of glycan structure verification can be gained through the use of exoglycosidases in these analytical workflows^3,4^.

**Figure 1:**
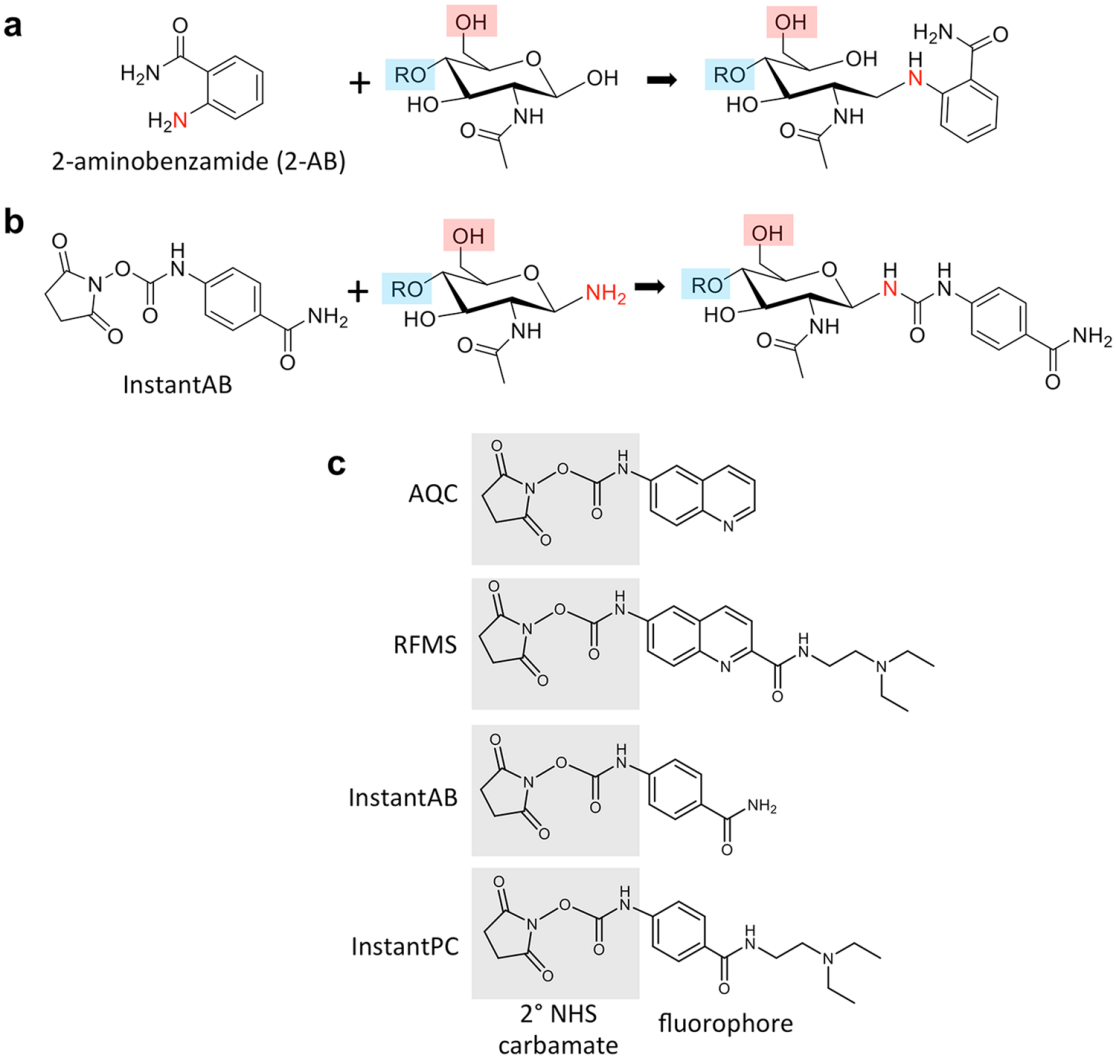
NHS-carbamate activated dyes and their attachment to the reducing end of *N-*glycans. (**a**,**b**) A comparison of the chemical linkage of two versions of 2-aminobenzamide (2-AB) to the reducing end GlcNAc of the chitobiose core of an *N-*glycan is shown (the blue highlighted R denotes the remaining portion of the *N-*glycan). (**a**) Standard 2-AB is added via reductive amination chemistry and creates an acyclic form of GlcNAc, whereas, (**b**) InstantAB is added via reactive NHS-carbamate chemistry and creates a urea linkage to a cyclic form of GlcNAc. In both panels, attachment of core fucose to the C6 position of GlcNAc is shown in pink highlight to illustrate its proximity to the label. (**c**) Structural features of the various NHS-carbamate activated dyes that are currently available for glycan analysis.

Over the past decade, numerous technical advances have significantly increased the speed, throughput, and sensitivity of *N-*glycan structural analyses^5^. This progress has been due to improvements in nearly every aspect of analytical workflow design. One significant workflow improvement has been the emergence of new fluorescent dyes for *N-*glycan analysis. Traditional amine-functionalized fluorescent dyes (*e.g*., 2-aminobenzamide [2-AB], 2-aminobenzoic acid [2-AA], procainamide [PC], and 8-aminopyrene-1,3,6-trisulfonic acid [APTS]) are each coupled to the reducing end GlcNAc of a free *N-*glycan using Schiff base chemistry (Fig. 1b). A new generation of dyes (*i.e*., RapiFluor-MS™ (RFMS))^6^, InstantAB™, InstantPC™, and 6-aminoquinolyl-*N-*hydroxysuccinimidylcarbamate (AQC)^7,8^ utilize reactive NHS-carbamate chemistry to form a covalent urea linkage between the fluorophore and a glycosylamine moiety that transiently resides on the reducing end GlcNAc following *N-*glycan release by PNGase F (Fig. 1a,c). This strategy significantly improves labeling speed, and the tertiary amines present on InstantPC and RFMS can also enhance the sensitivity of glycan detection in mass spectrometry applications^6^.

While newer labels have advantages for *N-*glycan analysis, an unexpected challenge has recently surfaced. The exoglycosidase bovine kidney fucosidase (BKF) has historically been the preferred enzyme for confirmation of the presence of α1-6 fucose side-branched to the *N-*glycan core. Recent studies reported that removal of core α1-6-linked fucose from *N-*glycans labeled with the aminoquinoline dyes AQC or RFMS by BKF was severely impeded^9,10^. Molecular modeling of BKF with a core fucosylated RFMS labeled *N-*glycan suggested that rigidity of the aminoquinoline label contributed to steric clashes within the BKF active site^9^. In the present study, we have extended these biochemical observations by showing that BKF is also inefficient at removing core α1-6-linked fucose from *N-*glycans labeled with NHS-carbamate derivatives of aminobenzamide dyes (*i.e*., InstantAB and InstantPC). Thus, BKF is severely limited as a tool for confirming *N-*glycan core fucose in workflows using any of the reactive NHS-carbamate fluorescent labels that are currently available.

We sought to identify a novel α1-6-fucosidase with the ability to remove core fucose from *N-*glycans labeled with reactive NHS-carbamate fluorescent dyes. We report here the cloning, expression and biochemical characterization of an α-fucosidase from *Omnitrophica* bacterium (termed fucosidase O). We show that fucosidase O has a strong preference for hydrolysis of α1-6-linked core fucose over α1-2-, α1-4-linked fucose in several glycans that we tested. The enzyme also can remove α1-3-linked core fucose that can occur on *N-*glycans of plants, worms and other non-mammalian eukaryotes. Finally, we demonstrate that fucosidase O is able to efficiently hydrolyze core α1-6-linked fucose on *N-*glycans labeled with NHS-carbamate activated fluorescent labels. Our study provides an alternative enzymatic solution for confirmation of *N-*glycan core α1-6 fucose.

## Results and Discussion

### Expression and purification of Omnitrophica α-L-fucosidase

A candidate α-L-fucosidase from *Omnitrophica* bacterium OLB16 (herein termed fucosidase O) was identified via computational interrogation of sequence repositories. Fucosidase O is a member of CAZy glycoside hydrolase family GH29. Its deduced protein sequence (KXK31601) shows 48% and 45% amino acid sequence identity to those of the bovine tissue (NP_001039500) and plasma (NP_001192747) α-L-fucosidases, respectively. Additionally, the enzyme has a 20 amino acid signal peptide as predicted by the SignalP 4.0 algorithm^11^. To generate recombinant protein for biochemical characterization, fucosidase O lacking its signal peptide was intracellularly expressed in *E. coli*. The recombinant protein was purified as described in Materials and Methods (see Supplementary Fig. S1). Finally, to ensure the accuracy of our specificity studies, purified fucosidase O was tested to assure the absence of any contaminating exoglycosidase activities using different fluorescent-labeled oligosaccharides (see Supplementary Fig. S2).

### Biochemical properties of fucosidase O

**Figure 2:**
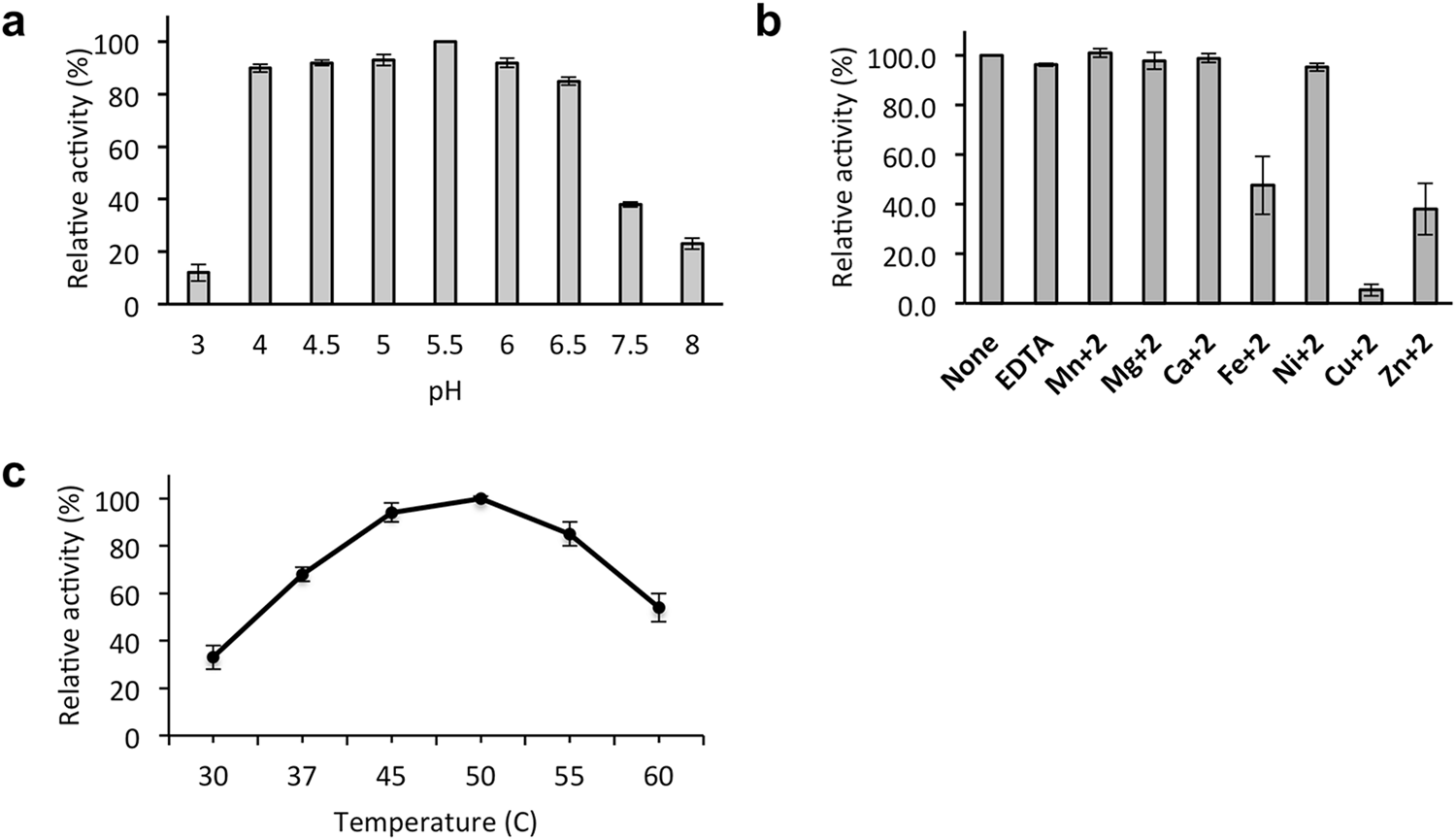
Biochemical properties of fucosidase O. The pH dependence (**a**), metal ion effect (**b**) and fucosidase activity at different temperatures (**c**) were determined. The 2-AB labeled core-fucosylated *N-*glycans M3N2F and NA2F were used as substrates to define the properties of fucosidase O. All experiments were performed in triplicate.

To define the optimal reaction conditions for fucosidase O, various biochemical properties of the enzyme were examined. Enzymatic activity was assessed from pH 3.0–8.0 using a trimannosyl *N-*glycan substituted with α1-6-linked core fucose as a substrate (see Supplementary Table S1). Fucosidase O was highly active from pH 4.0–6.0 with optimal activity at pH 5.5 (Fig. 2a). Fucosidase O was not affected by buffer containing Mn^2+^, Mg^2+^, Ca^2+^, or Ni^2+^ ions but showed significantly reduced activity in buffer containing Fe^2+^, Zn^2+^ or Cu^2+^ ions, with Cu^2+^ reducing activity by 95% under the reaction conditions used (Fig. 2b). The chelating agent EDTA had no effect on fucosidase activity, indicating that metal ions were not required for catalysis (Fig. 2b). The effect of temperature on enzyme activity and stability was also tested. The enzyme exhibited optimal activity at 50 °C (Fig. 2c).

### Substrate specificity of recombinant fucosidase O

**Figure 3:**
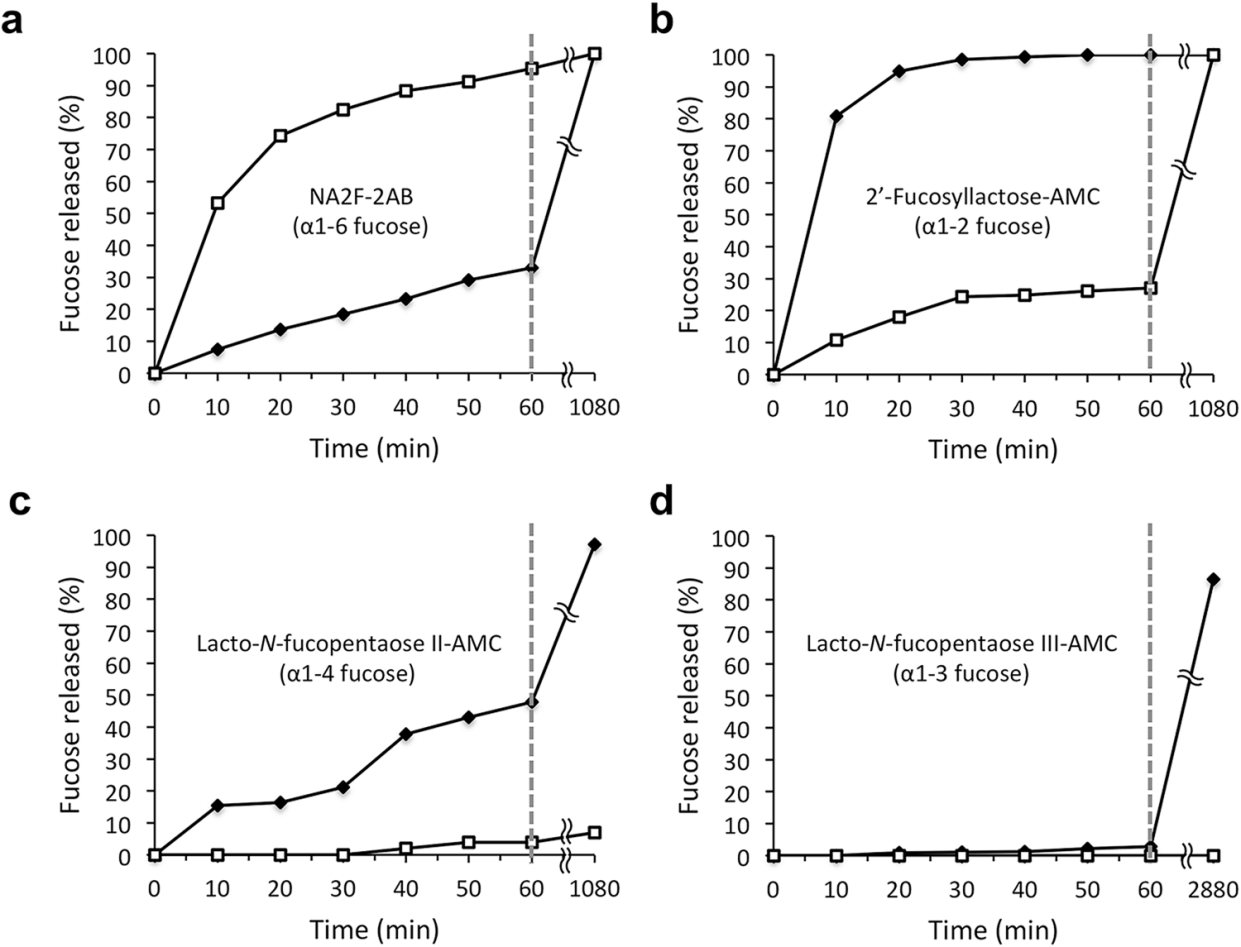
Substrate specificity and glycosidic bond preference of recombinant fucosidase O. Each oligosaccharide substrate (14 pmol) was mixed with fucosidase O or BKF (1.5 U/mL and 0.085 U/mL final concentration, respectively), and the reaction mixes were incubated at 37 °C. Aliquots were taken at each time point and glycans were analyzed by UPLC-HILIC-FLR. The chromatograms were integrated to measure the peak areas of the resulting glycans, and the percentage of fucose removal was calculated. Open squares indicate the glycans treated with fucosidase O; black diamonds indicate the glycans treated with BKF.

Substrate specificity of fucosidase O was tested using various fucosylated *N-*glycans and other oligosaccharides as substrates (see Supplementary Table S1). The substrates were fluorescently labeled with either 2-aminobenzamide (2-AB) or 7-amino-4-methylcoumarin (AMC). Each oligosaccharide substrate was mixed with the enzyme and incubated for 0–48 hours. Reaction mixtures were analyzed at different incubation time points by UPLC-HILIC-FLR. For each sample, the area of individual peaks corresponding to undigested and digested substrate was obtained via integration. This permitted calculation of the percent of released fucose from each substrate (see Materials and Methods). Complete digestion of α1–6-linked core fucose was observed with NA2F (an asialo-, galactosylated biantennary complex *N-*glycan with core fucose) (Fig. 3a). Terminal α1–2-linked fucose was also completely removed from 2-fucosyllactose (Fig. 3b). However, even after an extended 48 hour incubation, only 7% of α1-4-linked fucose was released from lacto-*N-*fucopentaose II (Fig. 3c), and no hydrolysis of α1-3-linked fucose was observed using lacto-*N-*fucopentaose III as a substrate (Fig. 3d).

The linkage preference of recombinant fucosidase O was compared to that of native BKF using fucosylated oligosaccharide substrates containing α1-2-linked fucose (2′-fucosyllactose) and α1-6-linked fucose (NA2F). After a 1 hour incubation, fucosidase O released α1-6 and α1-2-linked fucose with 95% and 27% efficiency, respectively; while BKF released 33% of α1-6-linked fucose and 100% of α1-2-linked fucose (Fig. 3a,b, dotted lines). After 18 hour incubation, both α1-6, α1-2-linked fucose substrates were completely hydrolyzed by both enzymes (Fig. 3a,b). Therefore, fucosidase O showed a marked preference for α1-6-linked core fucose (α1-6 >α1-2), whereas BKF preferred α1-2-linked fucose (α1-2 >α1-6).

Fucosidase O was also tested using fucosylated plant-derived *N-*glycans as substrates (see Supplementary Table S1). Glycans isolated from wild-type and genetically modified *Nicotiana benthamiana* plants^12^ were labelled with anthranilic acid (2-AA), and were incubated with fucosidase O. The reaction products were analyzed by MALDI-TOF-MS. No hydrolysis of the α1-3 fucosyl Lewis X motif attached to the *N-*glycan outer arm was detected (see Supplementary Fig. S4). However, both α1-3-linked core fucose and terminal α1-3 fucose attached to otherwise unsubstituted GlcNAc on the *N-*glycan outer arm were efficiently removed (see Supplementary Figs S3–S5). Thus, hydrolysis of α1-3-linked fucose by fucosidase O may vary greatly depending on the structural context and the substitution pattern of the GlcNAc residue to which the fucose is linked. Moreover, fucosidase O efficiently releases both α1-3- and α1-6-linked core fucose from a GlcNAc at the reducing end of *N-*glycans.

### Core α1-6 fucose removal from serum IgG N-glycans labeled with RapiFluor-MS (RFMS)

**Figure 4:**
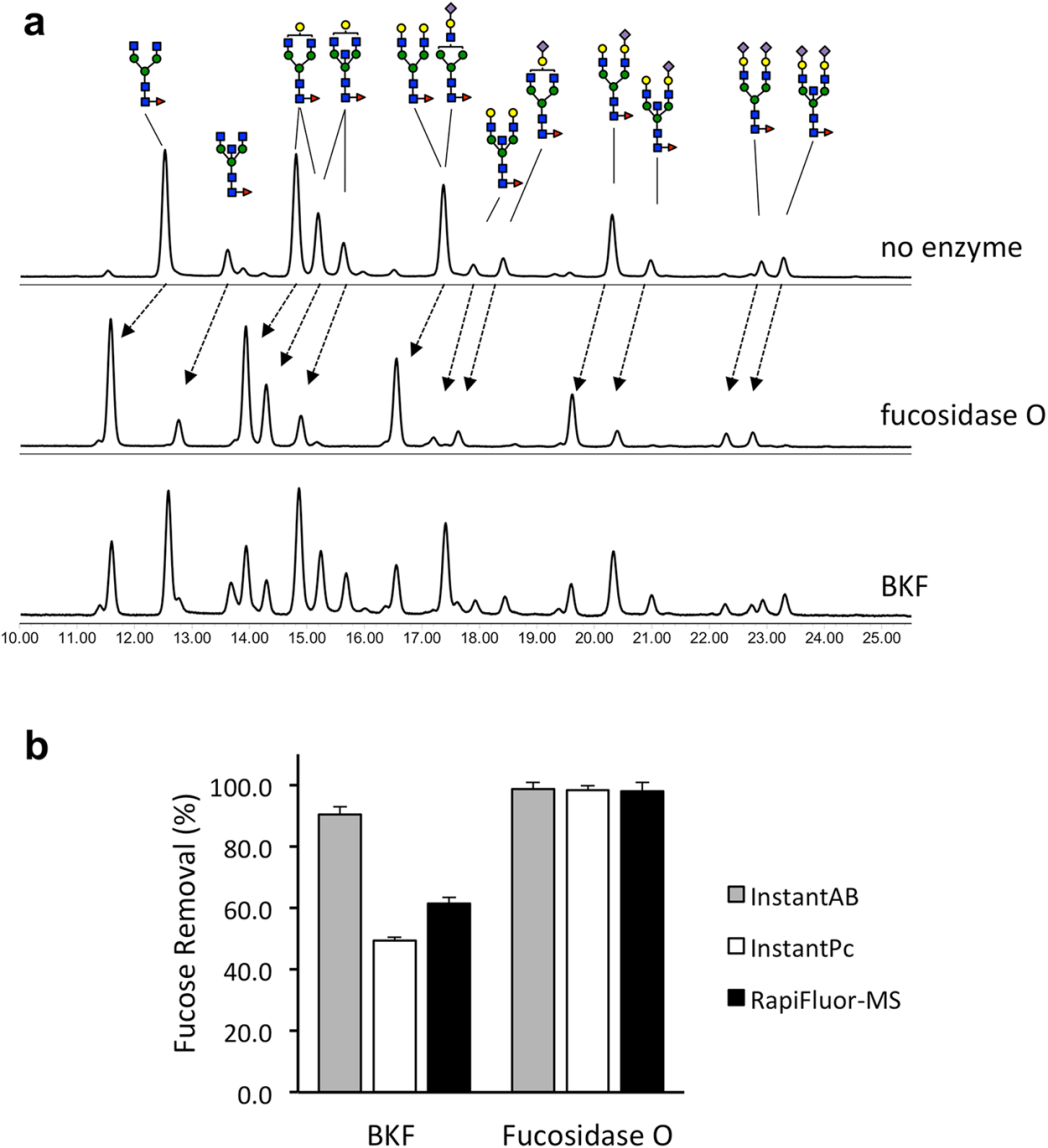
Core fucose removal from glycans labeled with different NHS-carbamate labels. (**a**) Complex *N-*glycans of human IgG labeled with RapiFluor-MS (8 pmol) were incubated with fucosidase O (35 U/mL) or BKF (2 U/mL) at 37 °C for 16 hours. After treatment, the glycans were analyzed by UPLC-HILIC-FLR. (**b**) Each *N-*glycan standard (2 pmol of NA2F-InstantAB, NA2F-InstantPC and NGA2F-RapiFluor-MS) was incubated with fucosidase O (35 U/mL) or BKF (2 U/mL) at 37 °C for 16 hours. The glycans were analyzed by UPLC-HILIC-FLR. The chromatograms were integrated to measure the peak areas of the resulting glycans, and the percentage of fucose removal was calculated. The experiments were performed in triplicate.

Fucosidase O was next tested for its ability to liberate core fucose from RFMS-labeled *N-*glycans in a complex sample. Human IgG harbors a mixture of many complex *N-*glycan structures, the vast majority of which are α1-6 core fucosylated^8,13^. In this experiment, an RFMS-labeled human IgG *N-*glycan mixture was used as a substrate (Fig. 4a). Only partial digestion of the core fucose on RFMS-labeled *N-*glycans was observed when treatment conditions were the same as those used for 2AB-labeled *N*-glycans (1.5 U/mL fucosidase O and 0.085 U/mL BKF). Thus, 23-fold higher concentration of fucosidase O (35 U/mL) and extended incubation time (16 hours) at 37 **°**C were required to achieve complete digestion of RFMS-labeled glycans (Fig. 4a). For comparison, a 16-hour reaction using a proportionally higher concentration of BKF (2 U/mL) showed only partial digestion and resulted in an increase in the sample’s complexity (Fig. 4a). Furthermore, increasing the amount of BKF in the reaction mixture to 5 U/mL and the length of digestion to 24 hours did not result in complete digestion (see Supplementary Fig. S6). This is consistent with the previously reported observation that RFMS hinders removal of core fucose by BKF^9^.

### Core α1-6 fucose removal from N-glycans containing urea-linked dyes

The efficiency of hydrolysis of α1-6-linked core fucose from an RFMS-labeled *N-*glycan (NGA2F) by BKF and fucosidase O was evaluated. Complete defucosylation of RFMS-labeled NGA2F was observed after treatment with fucosidase O (35 U/mL) for 16 hours at 37 °C (Fig. 4b). In comparison, digestion with BKF (2 U/mL) resulted in release of only ~61% core fucose from RFMS-labeled *N-*glycan (Fig. 4b). To expand on this observation, we also tested the ability of fucosidase O and BKF to remove core fucose from the *N-*glycan NA2F labeled with the urea-linked aminobenzamide dyes (InstantAB and InstantPC). This experiment was performed by incubating NA2F-InstantAB or NA2F-InstantPC with fucosidase O (35 U/mL) or BKF (2 U/mL) for 16 hours at 37 **°**C. BKF was able to remove 90% of core fucose from NA2F-InstantAB and only 49% from NA2F-InstantPC, whereas ≥99% of core fucose was released from both substrates by fucosidase O (Fig. 4b). These data illustrate that in addition to urea-linked aminoquinoline dyes, BKF can also be inhibited by urea-linked aminobenzamide dyes. In contrast, fucosidase O appears to efficiently remove core fucose in the presence of any of the existing urea-linked dyes.

## Summary and Conclusions

**Figure 5:**
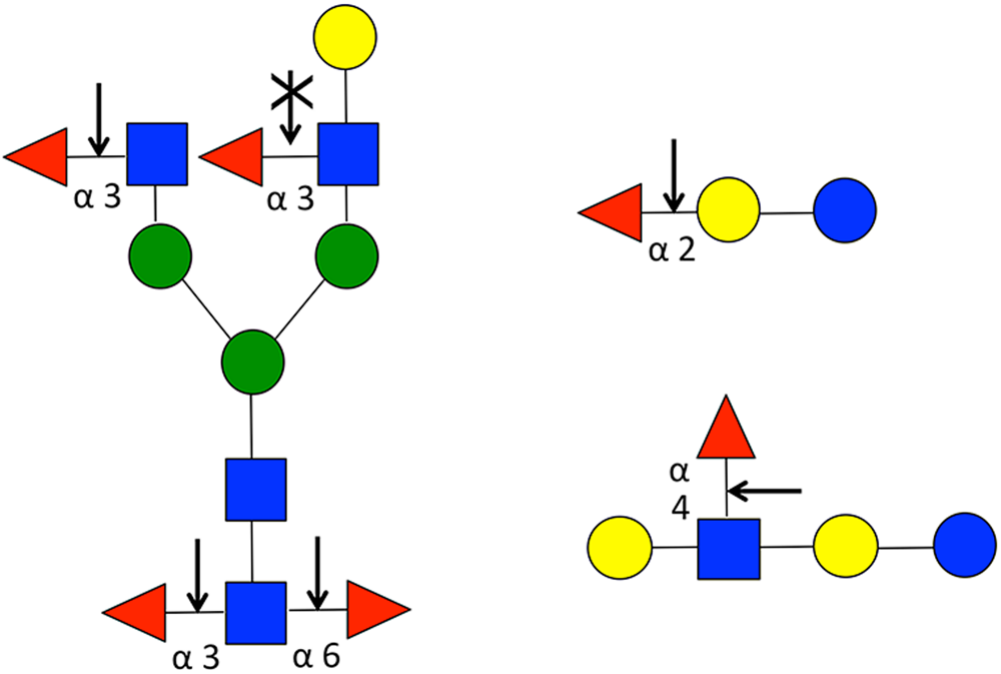
A schematic summary representation of recombinant fucosidase O specificity observed in this study. Observed cleavage of fucose from different positions of various *N*-glycans (left), 2′fucosyllactose (upper right) and lacto-*N*-fucopentaose (lower right) substrates is shown with black arrows. Outer arm α1-3 fucose was blocked when terminal galactose was present (arrow with X).

In this study, we identified a new fucosidase from *Omnitrophica* bacterium with the ability to efficiently remove core α1-6 fucose from *N-*glycans labeled with any of the newer reactive NHS-carbamate fluorescent dyes. In addition, due to its preference for α1-6 fucose, fucosidase O more efficiently removes core fucose from *N-*glycans labeled with traditional amide-linked labels compared to BFK. Furthermore, fucosidase O is also able to remove core α1-3 fucose from plant *N-*glycans but does not hydrolyze outer arm α1-3 fucose in the context of Lewis X, making this enzyme potentially important for discrimination of α1-3 fucose localization on *N*-glycans (Fig. 5). The novel specificity of fucosidase O improves upon the existing glycobiology toolbox and provides new options for enzymatic *N-*glycan structure confirmation.

## Methods and Materials

### Materials

All chemical reagents and solvents were purchased from Sigma-Aldrich. Labeled *N-*glycan substrates used for specificity tests and activity assays were obtained from Prozyme (Hayward, CA). A standard of human IgG *N-*glycans labeled with RapiFluor-MS was obtained from Waters (Milford, MA). Native bovine kidney fucosidase (GLYKO α(1-2,3,4,6) fucosidase) was from Prozyme. 2-chloro-4-nitrophenyl α-L-fucopyranoside (CNP-Fuc) was obtained from CarboSynth US (San Diego, CA).

### Cloning, expression and purification of fucosidase O

A DNA fragment (GenBank: LMZT01000142.1, Region: 29928-31277) encoding fucosidase O from *Omnitrophica* bacterium OLB16 (GenBank: KXK31601.1) was identified in the *Omnitrophica* genome database^14^. A codon optimized DNA sequence encoding fucosidase O lacking its signal peptide (23-449 amino acids) with terminal vector-specific overlapping sequences was synthesized by Integrated DNA Technologies (Coralville, IA). Bacterial expression vector pJS119^15^ was amplified using the primers 5′ ATGTTAACCTCCTAAGCTTAATTC 3′and 5′ GAATTCAGCTTGGCTGTTTTG 3′ and purified by gel extraction. The DNA fragment was cloned into pJS119 using the NEBuilder™ HiFi DNA Assembly Cloning Kit (New England Biolabs, Ipswich, MA). The resulting pJS119k-FucO plasmid and its sequence is available upon request.

For protein expression, the assembled plasmid pJS119k-FucO was introduced into *E. coli* NEB Express cells (New England Biolabs, Ipswich, MA). An overnight culture of transformed cells was diluted 1:100 in 2 L of LB medium supplemented with 50 μg/mL kanamycin and grown to 0.6 OD_600_ units at 37 °C. The expression of recombinant fucosidase O was induced by addition of isopropyl-β-thiogalactopyranoside (IPTG) to a final concentration of 0.4 mM with shaking for 4 h at 30 °C. The cells were harvested by centrifugation and resuspended in 50 mL of 20 mM Tris-HCl, pH 7.5. The cells were lysed by sonication with six 15 s bursts. Cell debris was removed by centrifugation at 19,000 × *g* for 60 min at 4 °C. The cleared cell lysate was applied to a 5 mL (bed volume) DEAE column (GE Healthcare Bio-Sciences AB, Uppsala, Sweden) equilibrated with 20 mM Tris-HCl, pH 7.5 at a flow rate of 2 mL/min. Bound proteins were eluted with a 0-300 mM NaCl gradient in 20 mM Tris-HCl, pH 7.5 and collected in 5 mL fractions. The fractions containing fucosidase O were pooled, ammonium sulfate was added to 1.5 M final concentration at 4 °C, and the protein sample was directly applied to a 5 mL (bed volume) Phenyl Sepharose FF (Low Sub) column (GE Healthcare Bio-Sciences AB, Uppsala, Sweden) at a flow rate of 3 mL/min. Bound protein was eluted using a reverse gradient of 1.5-0 M ammonium sulfate in 20 mM Tris-HCl, pH 7.5 (5 mL fraction size). Pooled fractions containing pure protein were dialyzed against 20 mM Tris-HCl, pH 7.5 containing 50 mM NaCl, 1 mM EDTA and concentrated using Vivaspin 20 concentrators (Sartorius Stedim Biotech, Göttingen, Germany). The yield of purified enzyme corresponded to 0.8 mg per liter of starting cell culture.

The colorimetric substrate 2-chloro-4-nitrophenyl α-L-fucopyranoside (CNP-Fuc) was used to assay enzymatic activity during purification. Typically, 1 µL α-fucosidase fraction was added to 100 µL of 2 mM CNP-Fuc in 20 mM sodium acetate buffer, pH 5.5, and incubated 1 hour at 37 °C. Light absorbance was read at 405 nm.

### Fucosidase O purity

Purified fucosidase O was tested for the presence of contaminating exoglycosidase activities using fluorescent-labeled oligosaccharides. Typically, fluorescent-labeled substrate (1 nmol) was incubated at 37 °C for 16 h with 3 μg of fucosidase O in 10 μL of reaction buffer (50 mM sodium acetate, pH 5.5). The reaction mix was spotted onto a silica-60 thin layer chromatography (TLC) plate (EMD Millipore, Gibbstown, NJ) and separated using a mobile phase of isopropanol-ethanol-water (110:50:25; v/v/v). Reaction products were visualized by UV light at 302 nm. Purified fucosidase O was tested for the following activities: β-*N-*acetylglucosaminidase, β-*N-*acetylgalactosaminidase, β-galactosidase, α-galactosidase, α-neuraminidase, α-mannosidase, α-glucosidase, β-xylosidase and β-mannosidase (see Supplementary Fig. S2).

### Fucosidase O unit definition and assay

One unit of fucosidase O was defined as the amount of enzyme required to cleave >95% of fucose from 1 nmol of the human IgG *N-*glycan G0F labeled with 2-aminoacridone (GlcNAcβ1-2Manα1-6(GlcNAcβ1-2Manα1-3)Manβ1-4GlcNAcβ1-4GlcNAc(Fucα1-6)-AMAC), in 1 hour at 37 °C in a total reaction volume of 10 µL. To assay fucosidase O, two-fold dilutions of enzyme were incubated with 1 nmol AMAC-labeled G0F substrate in 50 mM sodium acetate (pH 5.5) containing 5 mM CaCl_2_ in a 10 µL reaction. The reaction mix was incubated at 37 °C for 1 hour. Separation of reaction products were visualized via thin layer chromatography as described above.

### Fluorescent labeling of oligosaccharide substrates

To label *N-*glycan substrates with 2-aminobenzamide (2AB), 10 μL of a fluorescent labeling mix (350 mM 2AB, 1 M sodium cyanoborohydride in acetic acid/dimethyl sulfoxide [30:70]) was added to each tube containing a dried *N-*glycan sample. The reaction was incubated at 65 °C with agitation at 700 rpm for 120 min. For labeling with 7-amino-4-methylcoumarin (AMC), 10 μL of a labeling mix (430 mM AMC, 1 M sodium cyanoborohydride in acetic acid/methanol [4:30]) was added per 3 nmol of dried *N-*glycans. The reaction was incubated at 80 °C for 45 min. Excess label was removed by passage over HILIC SPE MacroSpin columns (Nest Group, Inc., Southborough, MA). Briefly, labeling samples (10 μL) were diluted to 300 μL with 90% acetonitrile/10% 50 mM ammonium formate, pH 4.4 (90% ACN/NH_4_F), loaded on columns equilibrated with the same buffer, and washed with 5 × 350 μL 90% ACN/NH_4_F. Samples were eluted in 100 μL 50 mM NH4F, pH 4.4.

### Determining biochemical properties of fucosidase O

To determine the optimum pH of fucosidase O, 2 pmol of 2AB-labeled trimannosyl N-glycan substituted with α1-6-linked core fucose (M3N2F) were incubated with 10 mU fucosidase O in parallel reactions containing 50 mM citrate buffer (pH 3.0), 50 mM sodium acetate buffer (pH 4.0-5.5), 50 mM 2-(*N*-morpholino)ethanesulfonic acid buffer (pH 6.0-6.5) or sodium phosphate buffer (pH 7.5–8.0). Each reaction had a final volume of 10 μL. After incubation for 30 min at 37 °C, 5 μL of each sample were analyzed by UPLC-HILIC-FLR.

To determine the optimum temperature fucosidase O, 2 pmol 2AB-labeled NA2F were mixed with 10 mU fucosidase O in 50 mM sodium acetate buffer, pH 5.5 containing 5 mM CaCl_2_ in a final reaction volume of 10 μL. Parallel identical reactions were incubated for 30 min at different temperatures (30-65 °C), after which 5 μL of each sample were analyzed by UPLC-HILIC-FLR.

To examine the effect of cations on fucosidase O activity, 2 pmol 2AB-labeled M3N2F were mixed with 10 mU fucosidase O in 50 mM sodium acetate buffer, pH 5.5 containing appropriate metal salts at the final concentration of 2 mM in a final reaction volume of 10 μL. After incubation for 60 min at 37 °C, 5 μL of each sample were analyzed by UPLC-HILIC-FLR.

### Substrate specificity of fucosidase O

Substrate specificity and the glycosidic bond preference of purified fucosidase O and BKF were compared using *N-*glycan or oligosaccharide substrates containing α1-6-, α1-2-, α1-3- or α1-4-fucose. A list of substrates used in this study is shown in Supplementary Table 1. Reactions consisted of 14 pmol of labeled glycan in 50 mM sodium acetate, pH 5.5 containing 5 mM CaCl_2_, and 105 mU of fucosidase O or 6 mU GLYKO α(1-2,3,4,6) fucosidase [BKF] in a total reaction volume of 70 µL (1.5 U/mL and 0.085 U/mL final concentration, respectively). Reactions were incubated at 37 °C and a 10 µL aliquot was harvested at regular time points. To each sample, 100 µL of 20% acetonitrile was added to stop the reaction. Each reaction mixture was transferred to the Nanosep 10 K Omega (Pall Life Sciences, Port Washington, NY) centrifugal device and centrifuged for 5 min at 12,000 × *g*. The samples were dried using a SpeedVac concentrator and were each dissolved in 10 μL of deionized water. For UPLC-HILIC-FLR analysis, 5 μL of each sample was mixed with 11.7 μL acetonitrile (final ratio 30:70 water/acetonitrile). An 10 μL aliquot of this mix was injected for UPLC-HILIC-FLR separation (next section).

Note that the activity units of each enzyme are defined using different substrates (*N-*glycan containing α1-6-linked fucose substrate used for fucosidase O, and a synthetic substrate 4-nitrophenyl α-L-fucopyranoside for BKF). Since the enzymes exhibit different activity on each of these two substrates, we used equimolar concentrations of each protein (0.15 μg) rather than equal number of units for comparison studies (see Supplementary Fig. S1).

### Glycan analysis by UPLC-HILIC-FLR

*N-*Glycans labeled with 2-AB or InstantAB were separated by UPLC using a Waters Acquity BEH glycan amide column (2.1 × 150 mm, 1.7 μm) on a Waters H-Class ACQUITY instrument (Waters Corporation, Milford, MA) equipped with a quaternary solvent manager and a fluorescence detector. Solvent A was 50 mM ammonium formate buffer pH 4.4 and solvent B was acetonitrile. The gradient was 0-1.47 min, 30% solvent A; 1.47-24.81 min, 30–47% solvent A; 25.5-26.25 min, 70% solvent A; 26.55-32 min, 30% solvent A. The flow rate was 0.56 mL/min. The injection volume was 10 μL and the sample was prepared in 70% (v/v) acetonitrile. Samples were kept at 5 °C prior to injection and the separation temperature was 40 °C. The fluorescence detection wavelengths were: λ_ex_ = 330 nm and λ_em_ = 420 nm for 2-AB; λ_ex_ = 278 nm and λ_em_ = 344 nm for InstantAB. The data collection rate was 20 Hz.

RapiFluor-MS-labeled human IgG *N-*glycans were separated by UPLC using a Waters Acquity BEH glycan amide column (2.1 × 150 mm, 1.7 μm) on a Waters H-Class ACQUITY instrument. Solvent A was 50 mM ammonium formate buffer pH 4.4 and solvent B was acetonitrile. The gradient used was 0-35 min, 25–46% solvent A; 36.5-39.5 min, 100% solvent A; 43.1-55 min, 25% solvent A. The flow rate was 0.4 mL/min. The injection volume was 5 μL and the sample was prepared in 75% (v/v) acetonitrile. Samples were kept at 5 °C prior to injection and the separation temperature was 60 °C. The fluorescence detection wavelengths were λ_ex_ = 265 nm and λ_em_ = 425 nm with a data collection rate of 20 Hz. Waters Empower 3 chromatography workstation software was used for data processing including traditional integration algorithm, no smoothing of the spectra and manual peak picking.

### Fucosidase O testing on plant N-glycans

Various *N-*glycans were isolated from recombinant glycoproteins produced in the wild-type and genetically modified *Nicotiana benthamiana* plants^12^. Isolation, labeling with anthranilic acid (2-AA) and characterization of these *N-*glycans were performed as described previously^12^.

The 2AA-labeled *N-*glycans were incubated overnight at 37 °C with 2 U of fucosidase O in 50 mM sodium acetate, pH 5.5 containing 5 mM CaCl_2_. After incubation, *N-*glycan products were loaded onto C18 Zip-Tip® (ZTC18M096, Merck Millipore, Amsterdam, The Netherlands) conditioned with 50% ACN + 0.1% TFA and then 0.1% TFA. The C18 resin was washed with 0.1% TFA and digestion products were eluted in 50% ACN + 0.1% TFA mixed with 2,5-dihydroxybenzoic acid (DHB) matrix (10 mg/ml, 8201346, Bruker Daltonics, Bremen, Germany) so that samples were directly spotted on a 384 well steel polished plate for MALDI analysis and dried at room temperature

2AA-labeled undigested and digested glycan samples were analyzed by Matrix Assisted Laser Desorption/Ionisation – Time of flight mass spectrometry (MALDI-TOF-MS) using an UltrafleXtrem® mass spectrometer (Bruker Daltonics) equipped with a 1 kHz Smartbeam II laser technology and controlled by the software FlexControl 3.4 Build 119 (Bruker daltonics). All spectra were recorded in the negative-ion reflectron mode using DHB as matrix. Bruker® peptide calibration mix (ref # 8206195) was used for external calibration. Spectra were obtained over a mass window of m/z 700 –3500 with ion suppression below m/z 700 for a minimum of 20,000 shots (2000 Hz) obtained by manual selection of “sweet spots”. The software FlexAnalysis 3.4 Build 76 was used for data processing including smoothing of the spectra (Savitzky Golay algorithm, peak width: m/z 0.06, 1 cycle), baseline subtraction (Tophat algorithm) and manual peak picking. For our purpose of highlighting fucosidase O specificity, only the major peaks in each spectrum were selected and peaks with a signal to noise ratio inferior to 3 were excluded as well as peak to which no glycan composition could be assigned. Deprotonated masses of the selected peaks were assigned using GlycoPeakfinder® tool of the software GlycoWorkbench 2 (www.glycoworkbench.org). The 2AA label was taken into account as a fixed reducing-end modification and possible glycan composition was set up based on the characterization work previously conducted by Wilbers *et al*.^12^ (i.e. 0-10 residues of deoxyhexose, hexose and N-acetylhexosamine and 0-1 pentose). A deviation of 150 ppm maximum was allowed for assignment of compositions.

## Electronic supplementary material

Supplementary Material
