# Supplementary material for: A novel broad specificity fucosidase capable of core α1-6 fucose release from N-glycans labeled with urea-linked fluorescent dyes
Source: Sci Rep. 2018 Jun 22;8:9504. doi: 10.1038/s41598-018-27797-0 (PMC6015026; doi:10.1038/s41598-018-27797-0)
Supplement: Supplementary file 1 — Supplementary Material [file 41598_2018_27797_MOESM1_ESM.docx]

**Supplementary Material**

**A novel broad specificity fucosidase capable of core α1-6 fucose release from *N*-glycans labeled with urea-linked fluorescent dyes**

Saulius Vainauskas^1^, Charlotte H. Kirk^1^, Laudine Petralia^1,2^, Ellen P. Guthrie^1^, Elizabeth McLeod^1^, Alicia Bielik^1^, Alex Luebbers^1^, Jeremy M. Foster^1^, Cornelis H. Hokke^2^, Pauline M. Rudd^3^, Xiaofeng Shi^1^, and Christopher H. Taron^1,^*

^1^ New England Biolabs, 240 County Road, Ipswich, MA 01938, USA

^2^ Department of Parasitology, Leiden University Medical Center, Albinusdreef 2, 2333 ZA Leiden, The Netherlands

^3^ NIBRT GlycoScience Group, National Institute for Bioprocessing, Research and Training, Foster’s Avenue, Mount Merrion, Blackrock, Co. Dublin, Ireland

*Corresponding author. Email: taron@neb.com

**Supplementary Table S1.** Glycan substrates used in this study.

| # | Motif | Name | Fluorescent Dye | Structure | Digestion by fucosidase O |
| --- | --- | --- | --- | --- | --- |
| 1. | α1-6 core fucose | NA2F | 2-AB  InstantAB  InstantPC  RapiFluor-MS | 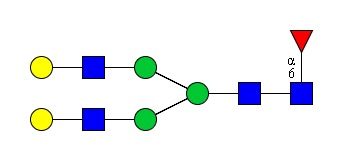 | Complete digestion |
| 2. | α1-6 core fucose | M3N2F | 2-AB | 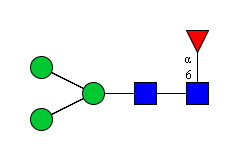 | Complete digestion |
| 3. | α1-6 core fucose | NGA2F | RapiFluor-MS | **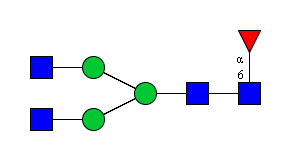** | Complete digestion |
| 3. | α1-3 core fucose | M3N2XF | 2-AA | 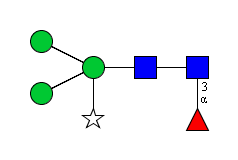 | Digested* |
| 4. | Lewis X | lacto-*N*-fucopentaose III | AMC | 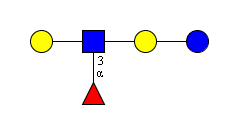 | No digestion |
| 5. | Lewis X | Fucosylated, xylosylated NA2 | 2-AA | 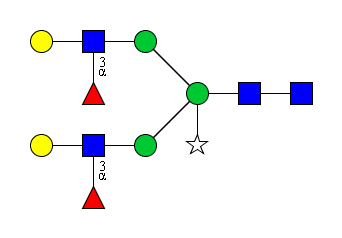 | No digestion* |
| 6. | Lewis A | lacto-*N*-fucopentaose II | AMC | 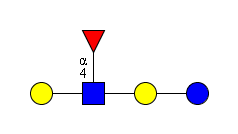 | Partially  digested |
| 8. | F-GlcNAc (terminal) | Xylosylated NGA2F | 2-AA | 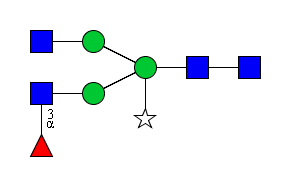 | Digested* |
| 9. | LDN-F | Xylosylated, GalNAc-containing NGA2F | 2-AA | 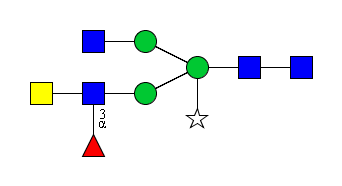 | No digestion* |
| 10. | 2'-Fucosyllactose | 2'-Fucosyllactose | AMC | 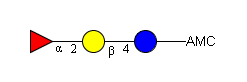 | Complete digestion |

* Digestion products of these *N*-glycans were analyzed by MALDI-TOF-MS

**A**

**B**

μg 0.15 0.3

mU 6 12 105 210

BKF

(native)

Fucosidase

O

BKF


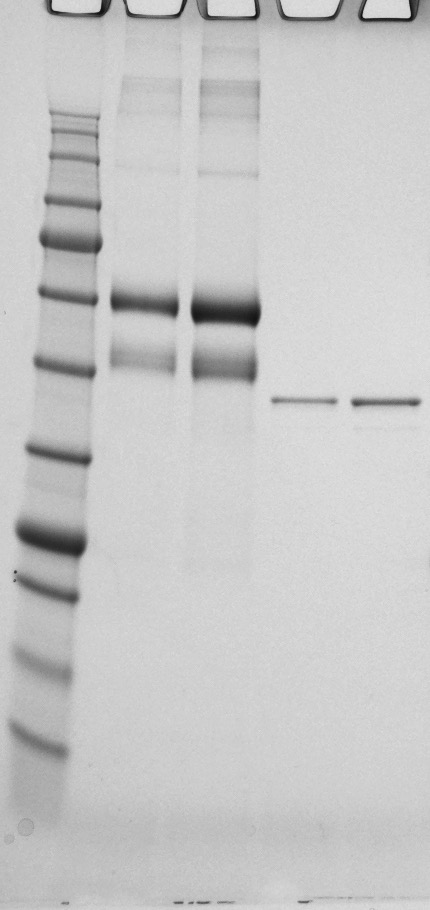


Fucosidase O

22

11

17

25

80

32

46

100

57

kDa

**C**

MYEPTWESLDSRPNPAWFDEAKFGIFIHWGVYAVPAWGSKGKYSEWYWNDMMDPNGETWKFHLKTYGEDFKYQDFAPMFKAEMFDPAQWADIFARSGAKYVVLTSKHHEGFCLWPSPDSWNWNSVDIGPHRDLCGDLTQAVRDRGLKMGFYYSLYEWFNPIYKTDVHRYVDQHMLPQLKDLVNRYQPSLIFSDGEWDHPSDVWRSTEFLAWLYNESPSREDVIVDDRWGKDTRGHHGGYYTTEYGNIYQAPEDAFQKRKWEECRGMGASFGYNRNETIDEYKPAGELIHLLIELVARGGNLLLDIGPTADGRIPVIMQQRLLEIGDWLKENGEGIYGSSPWRVNAEGDSVRYTTRDGAVYAHLLKWPGAELALESPKAGGTVEASLLGWPEPLACKVENGKIHISMPVIPPDNNTIRHAFVIRLKGVE

**Supplementary Figure S1.** (A) Analysis of fucosidase O by SDS-PAGE. Native bovine kidney fucosidase was loaded for comparison and titration. (B) Deconvoluted mass spectrum of the purified enzyme. The data was acquired on quadrupole-orbitrap (QExactive) mass spectrometer. The spectrum was analyzed using ProteinDeconvolution 4.0 software with ReSpect sliding window deconvolution algorithm. Reported deprotonated mass (49,382 Da) matches theoretical mass of the protein. (C) Amino acid sequence of recombinant fucosidase O expressed in *E. coli.*

**A**

| exoglycosidase activity | substrate |
| --- | --- |
|  |  |
| **1. β-N-Acetyl-glucosaminidase** | GlcNAcβ1-4GlcNAcβ1-4GlcNAc-AMC |
| **2. β-N-Acetyl-galactosaminidase** | GalNAcβ1-4Galβ1-4Glc-AMC |
| **3. β-Galactosidase** | Galβ1-3GlcNAcβ1-4Galβ1-4Glc-AMC |
| **4. β-Galactosidase** | Galβ1-4GlcNAcβ1-3Galβ1-4Glc -AMC |
| **5. α-Galactosidase** | Galα1-3Galβ1-4GlcNAc-AMC |
| **6. α-Galactosidase** | Galα1-6Galα1-6Glcα1-2Fru-AMC |
| **7. α-Neuraminidase** | Neu5Acα2-3Galβ1-3GlcNAcβ1-3Galβ1-4Glc-AMC |
| **8. α-Mannosidase** | Manα1-3Manβ1-4GlcNAc-AMC |
| **9. α-Mannosidase** | Manα1-6Manα1-6(Manα1-3)Man-AMC |
| **10. α- Glucosidase** | Glcα1-6Glcα1-4Glc-AMC |
| **11. β-Xylosidase** | Xylβ1-4Xylβ1-4Xylβ1-4Xyl-AMC |
| **12. β-Mannosidase** | Manβ1-4Manβ1-4Man-AMC |

**B**

**Supplementary Figure S2. (A)** Substrates used for testing of contaminating exoglycosidase activities. The reaction products were analyzed by TLC for digestion of substrate. (B) Fucosidase O activity was tested using G0F substrate (GlcNAcβ1-2Manα1-6(GlcNAcβ1-2Manα1-3)Manβ1-4GlcNAcβ-4GlcNAc(Fucα1-6)-AMAC) as described in Methods and Materials.

**
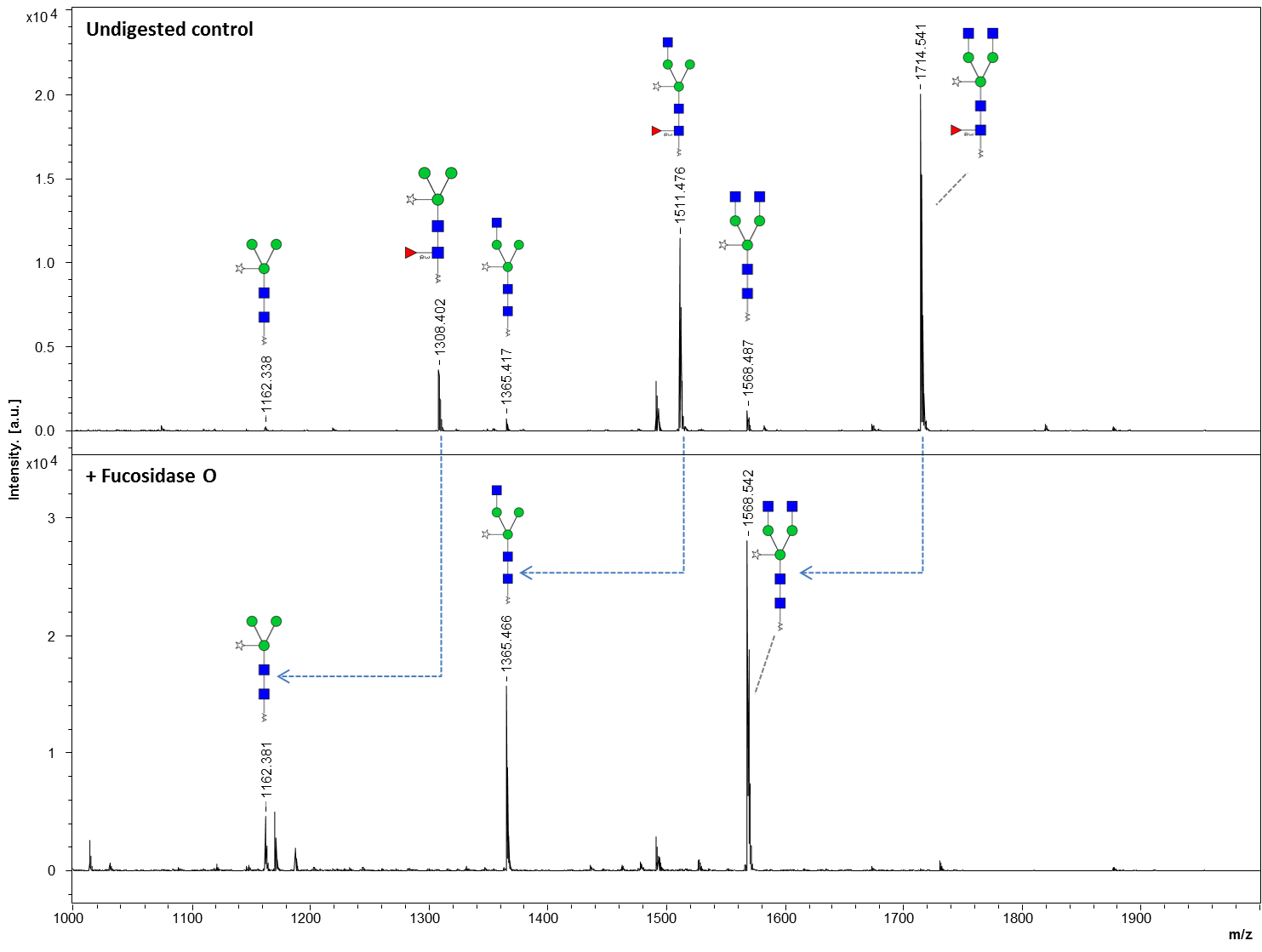
**

**Supplementary Figure S3.** Defucosylation of core α1-3 fucosylated N-glycans using fucosidase O. 2AA-labeled *N-*glycans derived from wild-type *N. benthamiana* plants were incubated overnight at 37°C with 2 U of fucosidase O and analyzed by MALDI-TOF-MS.

**
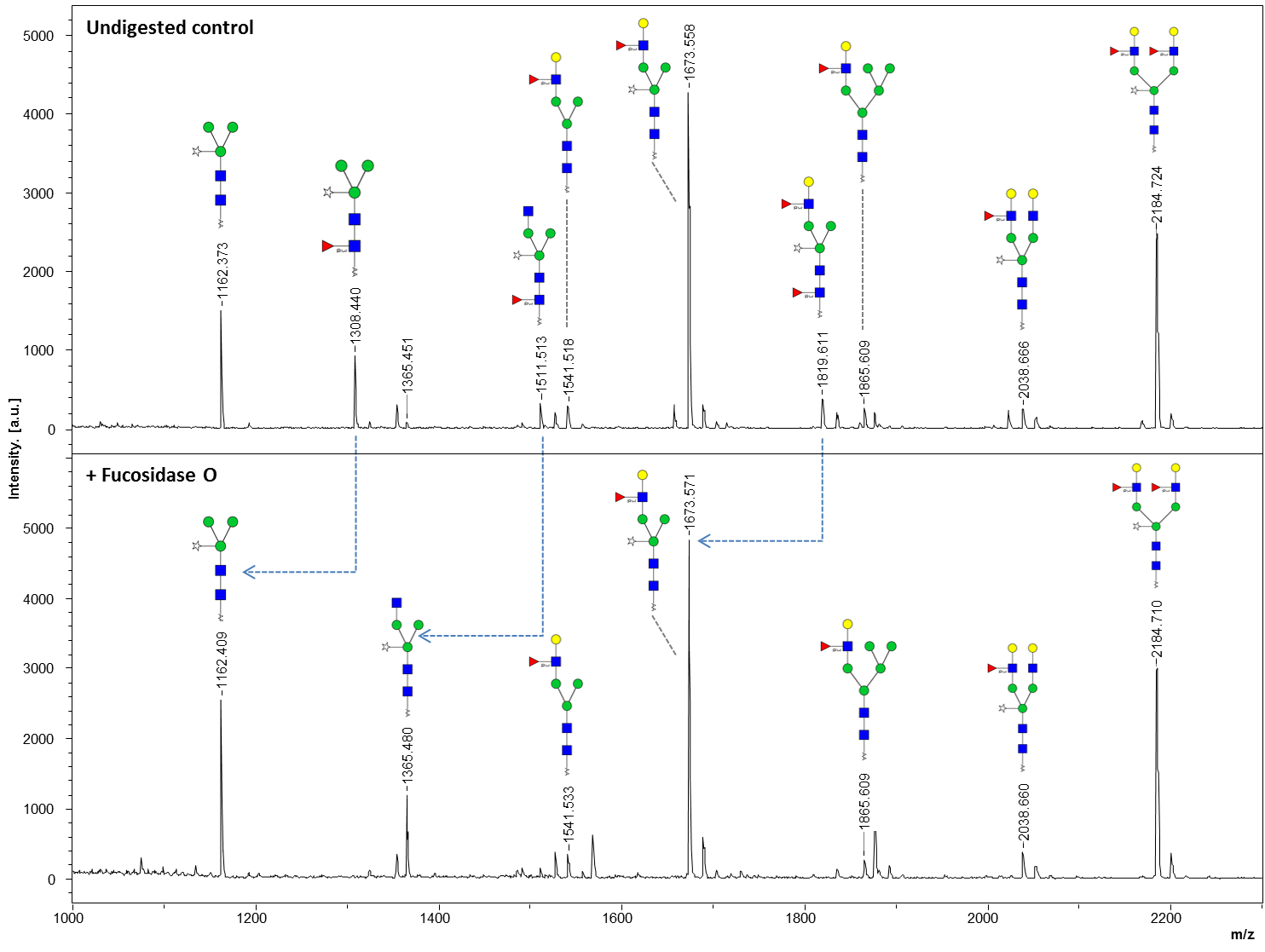
**

**Supplementary Figure S4.** Defucosylation of LeX-containing *N*-glycans using fucosidase O. 2AA-labeled *N*-glycans derived from LeX-engineered *N. benthamiana* plants were incubated overnight at 37°C with 2 U of fucosidase O and analyzed by MALDI-TOF-MS.


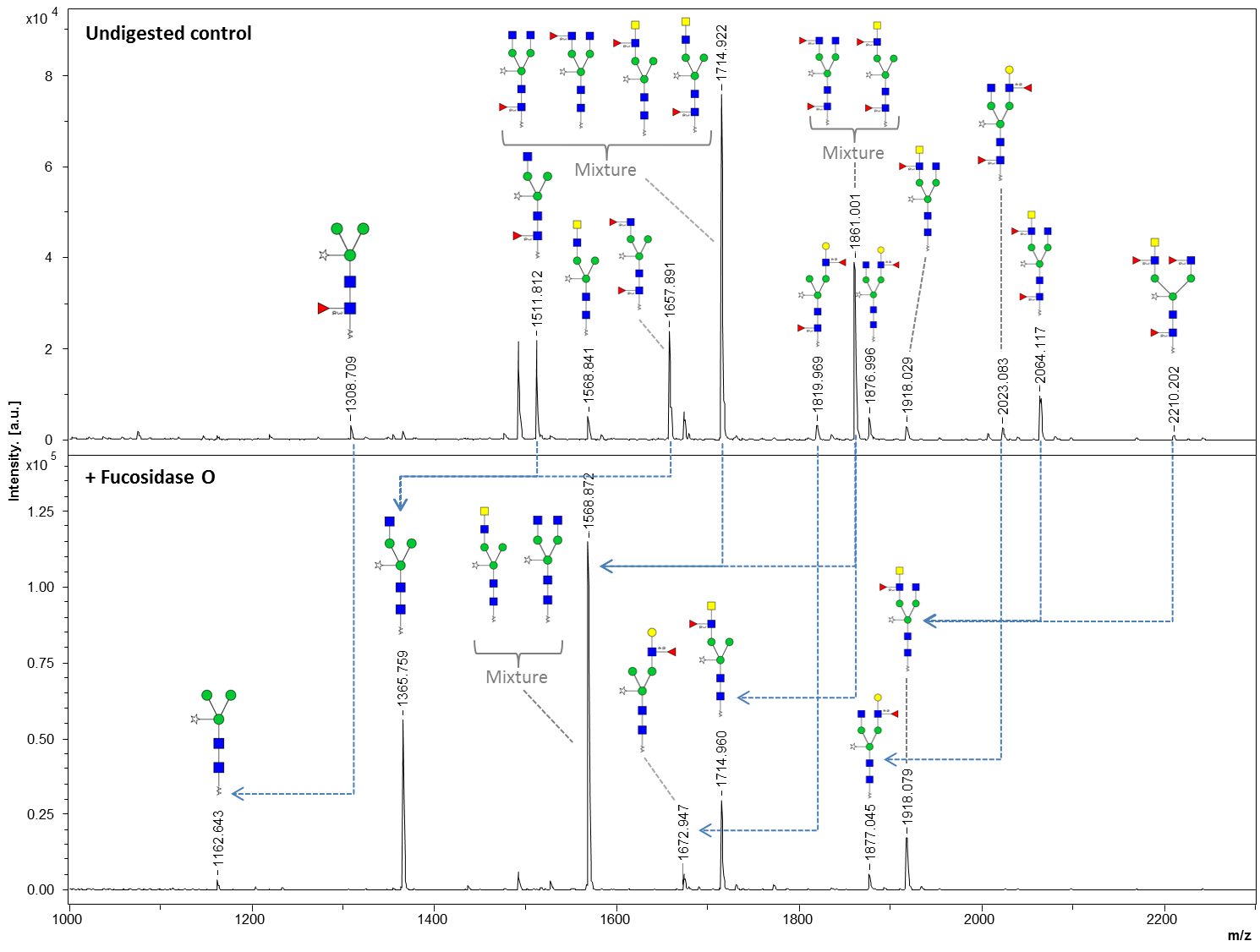


**Supplementary Figure S5.** Defucosylation of LDN-F containing *N*-glycans using fucosidase O. 2AA-labeled *N*-glycans derived from LDN-F-engineered *N. benthamiana* plants were incubated overnight at 37°C with 2 U of fucosidase O and analyzed by MALDI-TOF-MS.

*+ BKF*

*+ Fucosidase O*

*No enzyme*

**Supplementary Figure S6.** Defucosylation of different complex *N*-glycans of human IgG labeled with RapiFluor-MS. Labeled *N*-glycans (8 pmol) were incubated with fucosidase O (35 U/mL) or BKF (5 U/mL) at 37°C for 24 hours. After treatment, the glycans were analyzed by UPLC-HILIC-FLR.

**Supplementary Figure S7.** The pH dependence of fucosidase O. The enzyme activity was tested in different buffers using 2-AB labeled M3N2F substrate as described in Methods and Materials. The peak % areas of the reaction substrate and product were determined and the relative activities were calculated.

**Supplementary Figure S8.** Metal ion effect on fucosidase O activity. The enzyme activity was tested in the presence of different metals using 2-AB labeled M3N2F substrate as described in Methods and Materials. The peak % areas of the reaction substrate and product were determined and the relative activities were calculated.

**Supplementary Figure S9.**  Fucosidase O activity at different temperatures. The enzyme activity was tested using 2-AB labeled NA2F substrate as described in Methods and Materials. The peak % areas of the reaction substrate and product were determined and the relative activities were calculated.

**Supplementary Figure S10.** Defucosylation of NA2F *N*-glycan labeled with 2-AB . Labeled *N*-glycan (14 pmol) was incubated with fucosidase O (0.085 U/mL) or BKF (1.5 U/mL) at 37°C. After treatment, the glycans were analyzed by UPLC-HILIC-FLR. The peak % areas of the reaction substrate and product were determined.

**Supplementary Figure S11.** Defucosylation of 2-Fucosyllactose (2-FL) glycan labeled with AMC. Labeled glycan (14 pmol) was incubated with fucosidase O (0.085 U/mL) or BKF (1.5 U/mL) at 37°C. After treatment, the glycans were analyzed by UPLC-HILIC-FLR. The peak % areas of the reaction substrate and product were determined.

**Supplementary Figure S12.** Defucosylation of Lacto-*N*-fructopentaose II (LNFP II) glycan labeled with AMC. Labeled glycan (14 pmol) was incubated with fucosidase O (0.085 U/mL) or BKF (1.5 U/mL) at 37°C. After treatment, the glycans were analyzed by UPLC-HILIC-FLR. The peak % areas of the reaction substrate and product were determined.

 **Supplementary Figure S13.** Defucosylation of Lacto-*N*-fructopentaose III (LNFP III) glycan labeled with AMC. Labeled glycan (14 pmol) was incubated with fucosidase O (0.085 U/mL) or BKF (1.5 U/mL) at 37°C. After treatment, the glycans were analyzed by UPLC-HILIC-FLR. The peak % areas of the reaction substrate and product were determined.

**Supplementary Figure S14.** Core fucose removal from glycans labeled with different NHS-carbamate labels. Each *N-*glycan standard (2 pmol of NA2F-InstantAB, NA2F-InstantPC and NGA2F-RapiFluor-MS) was tested with fucosidase O or BKF as described in Methods and Materials. The peak % areas of the reaction substrate and product were determined. The appearance of an additional peak in the chromatogram (depicted by asterisk) is due to removal of core fucose from the different N-glycan.
